# Agriculture: Green Screen for Poultry Farms

**Published:** 2008-11

**Authors:** Lance Frazer

The Delmarva Peninsula, comprising Delaware and parts of Maryland and Virginia, is home to one of the country’s highest concentrations of poultry farms, some 2,000 outfits that can house upward of 75,000 birds at any one time. All those birds can mean a lot of dust, odor, and ammonia. At the August 2008 annual meeting of the American Chemical Society, researchers from the University of Delaware reported they have found a cost-effective way to help control these emissions.

Dust and odor are linked with respiratory effects in poultry workers, but little is known about the effects caused by these agents—beyond quality of life issues—among people who live near farms, conclude Dick Heederik and colleagues in the February 2007 issue of *EHP*. Of perhaps more immediate concern is the ammonia from poultry excrement. Numerous studies have linked atmospheric ammonia emissions to excessive algal growth in waters including the Chesapeake Bay. Ammonia can also combine with oxides of nitrogen and sulfur to form fine particulates small enough to lodge deep in human lungs.

In warm weather, poultry houses use “tunnel ventilation,” in which large fans pull air in one end and exhaust it—along with dust, feathers, and odors—out the other. In 2002, extension poultry specialist George Malone and his team planted a 30-foot-wide, three-row plot of trees as a so-called vegetative environmental buffer (VEB) opposite poultry house tunnel fans. The first row of trees was deciduous bald cypress, which caught the brunt of the dust and feathers. The second and third rows consisted of thickly needled Leyland cypress and Eastern red cedar, evergreens that acted as additional filters.

“We didn’t use evergreens in the first row, as the dust and feathers had a tendency to mat up on the leaves and could kill the tree,” Malone says. “With a deciduous tree, you get a fresh leaf screen every spring.” In measurements collected over six summers by sensors on each side of the VEB, the buffer reduced dust by 56%, ammonia by 53%, and odor by 18%.

Besides controlling emissions, VEBs also provide visual and auditory screens for farms as well as aid in energy efficiency by providing shade in summer and acting as windbreaks during the winter, says Malone. Moreover, their root systems absorb nutrients around the perimeter of the poultry houses, helping to reduce nitrogen runoff into surface and groundwater.

Penn State poultry scientist Paul H. Patterson and colleagues have studied a number of trees for use in VEBs. In an article in the January 2008 issue of the *Journal of Environmental Science and Health, Part B*, they reported that Spike hybrid poplar, ‘Streamco’ purpleosier willow, and hybrid willow were effective at removing ammonia emissions from the air around poultry farms, while Norway spruce and hybrid willow were the best at trapping dust and associated odors.

To date, about one-third of the Delmarva poultry farms have established VEBs. Up to 75% of the cost of VEBs may be covered under programs such as the Environmental Quality Incentives Program of the Natural Resources Conservation Service.

Bill Rohrer, a program administrator for Delaware’s Nutrient Management Commission, says, “The VEB concept that Malone has developed is an excellent step in emissions reduction. We’ve seen that fifty percent of all ammonia that comes out of the building is deposited within a few hundred yards of the farm, so if you build the vegetative screen where Malone’s research has put it, you can do an excellent job of cutting back on atmospheric ammonia and dust.”

“This isn’t a silver bullet,” Malone says. “But it’s a green, cost-effective technology for an industry that makes its living on being cost-effective.”

